# Developing a point-of-care electronic medical record system for TB/HIV co-infected patients: experiences from Lighthouse Trust, Lilongwe, Malawi

**DOI:** 10.1186/s13104-016-1943-4

**Published:** 2016-03-05

**Authors:** Hannock Tweya, Caryl Feldacker, Oliver Jintha Gadabu, Wingston Ng’ambi, Soyapi L. Mumba, Dave Phiri, Luke Kamvazina, Shawo Mwakilama, Henry Kanyerere, Olivia Keiser, Johnbosco Mwafilaso, Chancy Kamba, Matthias Egger, Andreas Jahn, Bertha Simwaka, Sam Phiri

**Affiliations:** The International Union Against Tuberculosis and Lung Disease, Paris, France; Lighthouse Trust, Lilongwe, Malawi; Institute of Social and Preventive Medicine (ISPM), University of Bern, Bern, Switzerland; International Training and Education Center for Health (I-TECH), University of Washington, 901 Boren Avenue, Suite 1100, Seattle, WA 98104 USA; Baobab Health Trust, Lilongwe, Malawi; Department of Biomedical Informatics, Center for Health Informatics for the Underserved, University of Pittsburgh, Pittsburgh, PA USA; Malawi National Tuberculosis Program, Lilongwe, Malawi; Bwaila District Hospital, TB clinic, Lilongwe, Malawi; Central Monitoring and Evaluation Division/Department for HIV and AIDS, Ministry of Health, Lilongwe, Malawi

**Keywords:** Electronic medical records, TB/HIV integration, Malawi, Informatics, Monitoring and evaluation

## Abstract

**Background:**

Implementation of user-friendly, real-time, electronic medical records for patient management may lead to improved adherence to clinical guidelines and improved quality of patient care. We detail the systematic, iterative process that implementation partners, Lighthouse clinic and Baobab Health Trust, employed to develop and implement a point-of-care electronic medical records system in an integrated, public clinic in Malawi that serves HIV-infected and tuberculosis (TB) patients.

**Methods:**

Baobab Health Trust, the system developers, conducted a series of technical and clinical meetings with Lighthouse and Ministry of Health to determine specifications. Multiple pre-testing sessions assessed patient flow, question clarity, information sequencing, and verified compliance to national guidelines. Final components of the TB/HIV electronic medical records system include: patient demographics; anthropometric measurements; laboratory samples and results; HIV testing; WHO clinical staging; TB diagnosis; family planning; clinical review; and drug dispensing.

**Results:**

Our experience suggests that an electronic medical records system can improve patient management, enhance integration of TB/HIV services, and improve provider decision-making. However, despite sufficient funding and motivation, several challenges delayed system launch including: expansion of system components to include of HIV testing and counseling services; changes in the national antiretroviral treatment guidelines that required system revision; and low confidence to use the system among new healthcare workers. To ensure a more robust and agile system that met all stakeholder and user needs, our electronic medical records launch was delayed more than a year. Open communication with stakeholders, careful consideration of ongoing provider input, and a well-functioning, backup, paper-based TB registry helped ensure successful implementation and sustainability of the system. Additional, on-site, technical support provided reassurance and swift problem-solving during the extended launch period.

**Conclusion:**

Even when system users are closely involved in the design and development of an electronic medical record system, it is critical to allow sufficient time for software development, solicitation of detailed feedback from both users and stakeholders, and iterative system revisions to successfully transition from paper to point-of-care electronic medical records. For those in low-resource settings, electronic medical records for integrated care is a possible and positive innovation.

**Electronic supplementary material:**

The online version of this article (doi:10.1186/s13104-016-1943-4) contains supplementary material, which is available to authorized users.

## Background

As the numbers of patients co-infected with human immunodeficiency virus (HIV) and tuberculosis (TB) increase in sub-Saharan Africa, reliance on paper-based registers may result in poor record management and, ultimately, suboptimal clinical care. One innovative approach to improve data quality and strengthen the continuum of care is the implementation of user-friendly, real-time electronic medical record (EMR) system. EMR systems may be well received by clinicians [1], improve the quality of clinic data [2], and increase the quality of patient care by ensuring adherence to clinical guidelines and reducing errors [3, 4].

In 2005, Lighthouse Trust, the largest public provider of antiretroviral therapy (ART) and TB services in Malawi’s central region, in partnership with Baobab Health Trust, a local non-governmental organisation that leads the development of EMR system in the country, developed and launched an ART-focused EMR system. By 2006, both of Lighthouse Trust’s clinics, Lighthouse and Martin Preuss centre (MPC), successfully employed the real-time, touchscreen-based EMR system to manage ART patients. Data management for more than 36,000 ART patients at both clinics is now entirely based on the EMR system resulting in near completeness of patient level data. Due to the EMR system success in improving both patient management and reliable record keeping, described previously [5], the Ministry of Health (MoH) formally adopted and implemented Baobab’s EMR system in high burden ART sites, scaling up to 39 ART facilities with more than 2500 patients by 2013 [6]. At national level, monitoring and evaluation of the ART program, including timely generation of ART supervision reports, improved dramatically as a result of the ART EMR system roll-out.

Although the current ART EMR system in Malawi is successful, one significant limitation is its singular programmatic focus. Nationally, almost 60 % of TB patients are co-infected with HIV [7]. At MPC clinic, Lighthouse’s integrated TB/HIV clinic run in partnership with the District Health Office (DHO) [8], almost 50 % of its 3500 TB suspects and 60 % of 3200 TB patients are TB/HIV co-infected. These co-morbidities resulted in information for TB/HIV patients being stored in dual record systems: TB-related information for TB suspects and TB patients was recorded in paper registers while ART data for these same patients were recorded in the ART EMR system. Subsequently, paper data for all TB and TB/HIV co-infected patients were entered retrospectively in an off-line Microsoft Access database; data for those co-infected was also linked to ART EMR system information using unique patient identifiers. This dual-record system created immense challenges as paper-based TB registers were often incomplete with some treatment and follow-up information missing, thus creating barriers in TB/HIV service integration, reducing program quality, and diminishing data quality for program monitoring.

In 2010, Lighthouse Trust, with its partners Baobab Health Trust, the Malawi National TB Program (NTP), and the IeDEA Collaboration at the Institute of Social and Preventive Medicine at University of Bern in Switzerland, received a one-year innovation development grant from US President’s Emergency Plan for AIDS Relief (PEPFAR) to develop and implement a real-time, touchscreen-based EMR system for TB and TB/HIV integrated care. The TB/HIV EMR system is expected to significantly increase service quality and facilitate program monitoring at MPC. If proven beneficial, the system will expand from MPC to other clinics.

This paper shares insights and lessons learned from our three-phased approach to planning, developing, and implementing the point-of-care TB/HIV EMR system for TB and TB/HIV co-infected patient management implemented at MPC clinic. We first detail the steps taken in conjunction with developers, system users and local stakeholders during system planning. Then, we note the system components designed to aid patient care followed by our experiences in system deployment. Then, we discuss the delays and unexpected challenges we overcame during implementation. These experiences and lessons may assist providers, clinics and organizations in other resource limited settings that are considering developing and deploying TB/HIV EMR systems.

### Ethics statement

The Malawi national Health Science Research Committee (NHSRC) provided a general blanket approval for the use of routine programmatic data for monitoring and evaluation every year; and as such, a separate formal ethics approval was not required from NHSRC.

### Availability of supporting data

The TB/HIV EMR system and its components are Open Source. The code and system components are available for non-commercial, scientific use at https://github.com/baobabhealthtrust/TB-ART.

### Phase I: Engaging multiple stakeholders in TB/HIV EMR system planning and development

#### Building ownership and buy-in

While the technical processes were being discussed with management and technical staff at Lighthouse and Baobab, a series of concurrent meetings were conducted by Lighthouse staff with Baobab system developers, DHO TB staff, NTP, and MoH’s HIV department. Due to the rapid nature of the award and expected results, these initial, highly-participative meetings were held over the course of several weeks, helping ensure buy-in, create ownership, and discuss perceived barriers to TB/HIV EMR implementation (Project timeline: Additional file 1: Figure S1). These meetings allowed all key stakeholders to take part in the planning process and come to consensus on a plan of action for a participatory process of system development, testing, and implementation.

#### Mapping efficient patient flow

For the successful design and deployment of a TB/HIV EMR system, it is critical to correctly analyze and map patient flows to mimic or improve upon standardized patient-provider interactions throughout various clinic visit types and patient services. At MPC, the majority of patients who needed ART only were already efficiently moving through the ART EMR process from registration, weight/height, clinic review, laboratory (if needed), pharmacy, and exit. Unlike the simpler flow for ART patients who largely follow the same visit flow, most TB suspects and patients fell under one of five, distinct, patient-provider encounter types depending on HIV status, TB suspects, TB patients (smear positive results, no x-rays), follow-up TB patients already in care with unknown HIV status or not yet on ART, follow-up patients on ART at MPC, and transfer-in TB patients (Illustrative patient flow diagrams: Additional file 2: Figure S2). To determine the flow for these different TB/HIV patient types in this clinic, we used a mix of interviews with key staff and observational assessments to understand patient flow, and found that unnecessary complications and frequent redundancies led to confusion among providers and long waiting times for patients. For example, patients previously needed to see different clinicians to meet their TB and ART needs, passing through registration to document each step in their visit before proceeding. Therefore, instead of creating a TB/HIV EMR system that mimicked the existing flow, we needed to first implement systematic changes in patient flow to facilitate efficient and timely care that could be applied to MPC and other integrated clinics. Over a 6 month period, proposed patient flows were further discussed, tested, and modified through a series of observation assessments, mapping exercises, and consultative meetings with providers to ensure that the flows would reflect the continuum of care services including all testing, treatment, follow-up, prescription, and referral components. At the end of the mapping process, the final seven patient flows were used to draft logical flow charts of services to develop the TB/HIV EMR system components that would be applicable in MPC and other integrated clinics to optimize care.

#### Strengthening monitoring and evaluation

Routine, accurate reporting of key service quality indicators among TB and TB/HIV patients is complicated by the need to aggregate patient level information from multiple electronic and paper systems [9]. Prior to the development and launch of the TB and TB/HIV EMR, paper-based data for TB patients and TB/HIV patients was entered manually, taking several hours each week. Therefore, all stakeholders agreed that the new system must simplify this process while improving the quality of the data similar to how the ART EMR helped streamline patient data collection and improved data quality over time for HIV-infected patients. The TB/HIV EMR system has built-in capacity to generate quarterly reports that correspond to the national TB and ART/prevention of mother to child transmission (PMTCT) program indicators and guidelines. Data collected by the TB/HIV EMR would then be used to gauge progress against targets, identify weaknesses in patient care, and plan for improved service delivery at the facility and national levels.

#### Addressing technology challenges

Since many EMR systems require both specific hardware and computer literacy among users, low resource settings often encounter significant barriers. To address these challenges, our hardware is based on real-time, touchscreen systems that are easy to use as demonstrated by more than 10 years of successful utilization in Malawi [5].To protect patient information and reduce risk of computer viruses, the TB/HIV EMR computers do not permit internet access or other operations.

Sustainable electrical power, essential for the TB/HIV EMR system, also presented challenges. We employed rechargeable batteries and inverters that could support the system for 48 hours in case of power cuts. For long power outages, a stand-by generator comes in. For longer power cut or system failure issues, the paper-based back-up system provides additional support to maintain patient services.

### Phase II: Developing TB/HIV EMR system components and functionalities

#### General system description

**Fig. 1:**
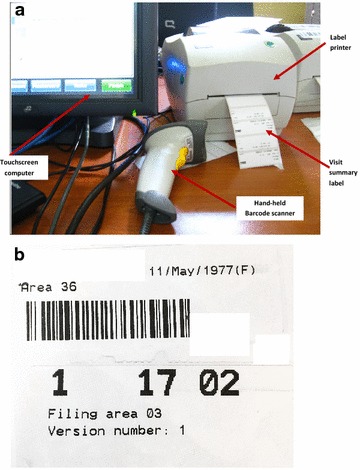
**a** Work station for TB/ART Electronic Medical Record system at at Martin Preuss centre in Lilongwe, Malawi. **b** Stickers for unique patient id and filing number for the TB/ART Electronic Medical Record system at Martin Preuss centre in Lilongwe, Malawi

**Fig. 2:**
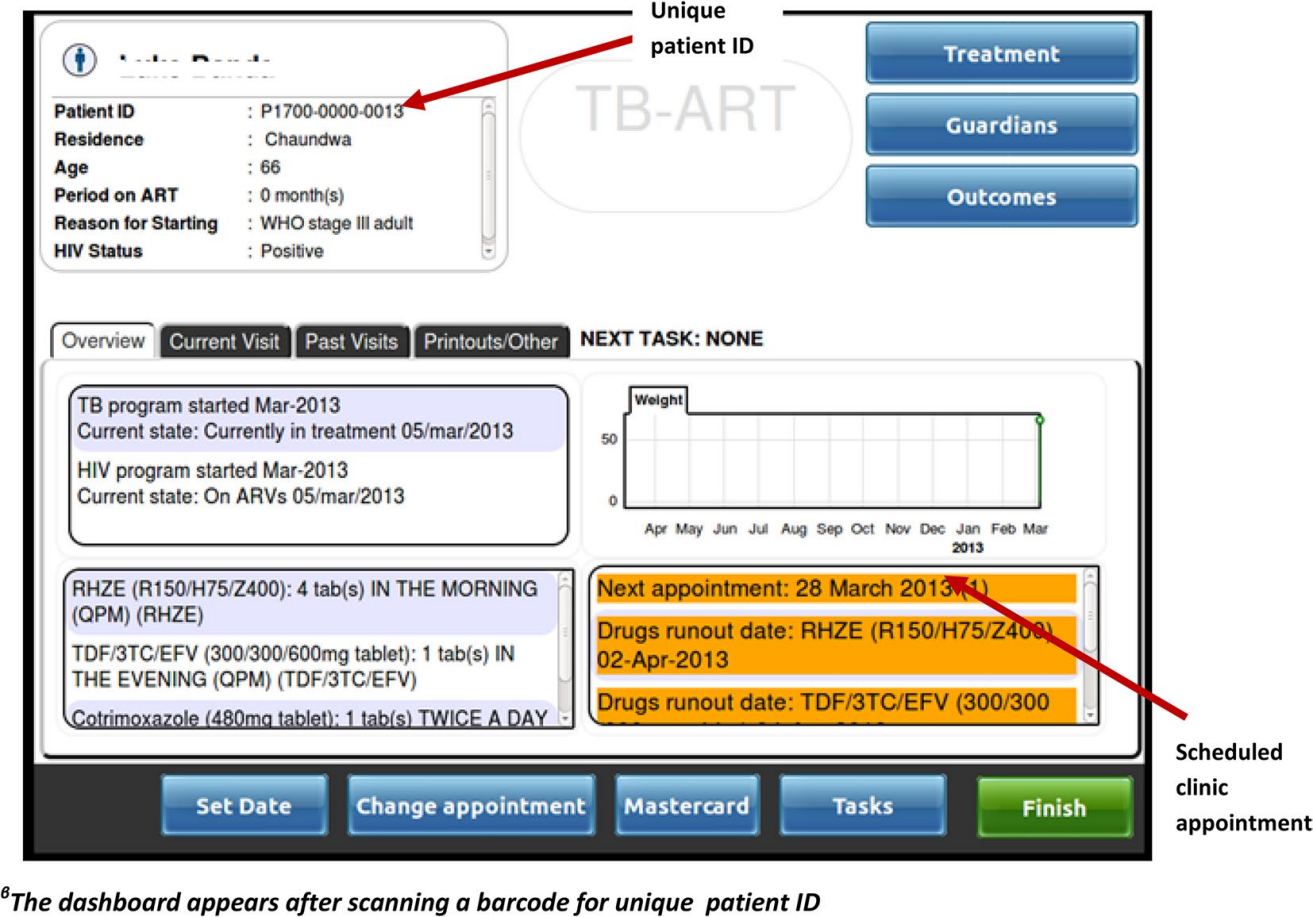
A dashboard for the TB/ART Electronic Medical Record system at at Martin Preuss centre in Lilongwe, Malawi

As with the ART EMR, the TB/HIV EMR system is a real-time, point-of-care, intranet-based system based on open source software that employs the MySQL OpenMRS data model [10]. Key TB/HIV EMR system components include: registration of demographics, anthropometric measurements, management of laboratory samples and results (Smear microscopy of sputum, CD4 count, viral load etc.), HIV testing, TB diagnosis, WHO HIV clinical staging, family planning services, TB/HIV clinical review and drug dispensing. Other co-morbidities and infections are also documented during clinical review including diabetes, cancer, meningitis, and malaria. The TB/HIV EMR system uses touchscreen technology with terminals in reception and clinical consultation rooms. All TB- and HIV-related information is available through the same touchscreen at the terminal. Each terminal is equipped with a hand-held barcode scanner and a small printer, issuing unique patient id, filing number and duplicative visit summary stickers (labels) for patient files and health passports (Fig. 1a, b). Through the intranet, each terminal is connected to a central server where data from ART EMR and TB/HIV EMR modules are stored in the same database. The data are stored and backed-up nightly, on-site and off-site. The terminals do not provide access to internet or other software applications. Figure 2 shows the dashboard (first page) of the system.

#### Improving management of patient files

With high patient numbers, manual management of paper files for TB/HIV patients is a challenge. Currently, during patient registration at MPC, each TB suspect or patient is assigned a unique patient ID, which is placed on their health passport and paper file and a filing number, which is used to locate the paper file in the shelving system. Older dormant files (files for patients who are lost to follow-up, transfer to other clinic or dead) are moved to archive areas; the process of identifying the longest dormant file is automated in the TB/HIV EMR system. As patient numbers continue to increase, filing space will run out, leaving the potential for missing or misplaced files. To address this challenge, the TB/HIV EMR system reduces the need for complete paper files; however, the system does retain capacity for generating printed summary files for patient who transfer. As more ART facilities adopt TB/HIV EMR technology for ART and TB/HIV services, we expect the use of paper-based records to stop.

#### Ensuring compliance with ART and TB guidelines

Compliance with guidelines can present challenges especially with complicated patients. The TB/HIV EMR system is designed to enforce compliance to national TB and ART/PMTCT guidelines by requiring clinicians to complete key tasks such as ascertainment of HIV status and ART status, as needed. The user interface software was designed to reduce system complexity by presenting only one question and possible answers on one screen page, helping to reduce user error and ensure data completeness. The questions are simple and concise. Each question requires an answer to progress. The system prompts healthcare providers to consider additional prevention and treatment tasks during reviews, including ordering lab samples (sputum, viral load etc.), consider drug contraindications, and counsel on drug adherence.

#### Improving drug dispensing and accountability

**Fig. 3:**
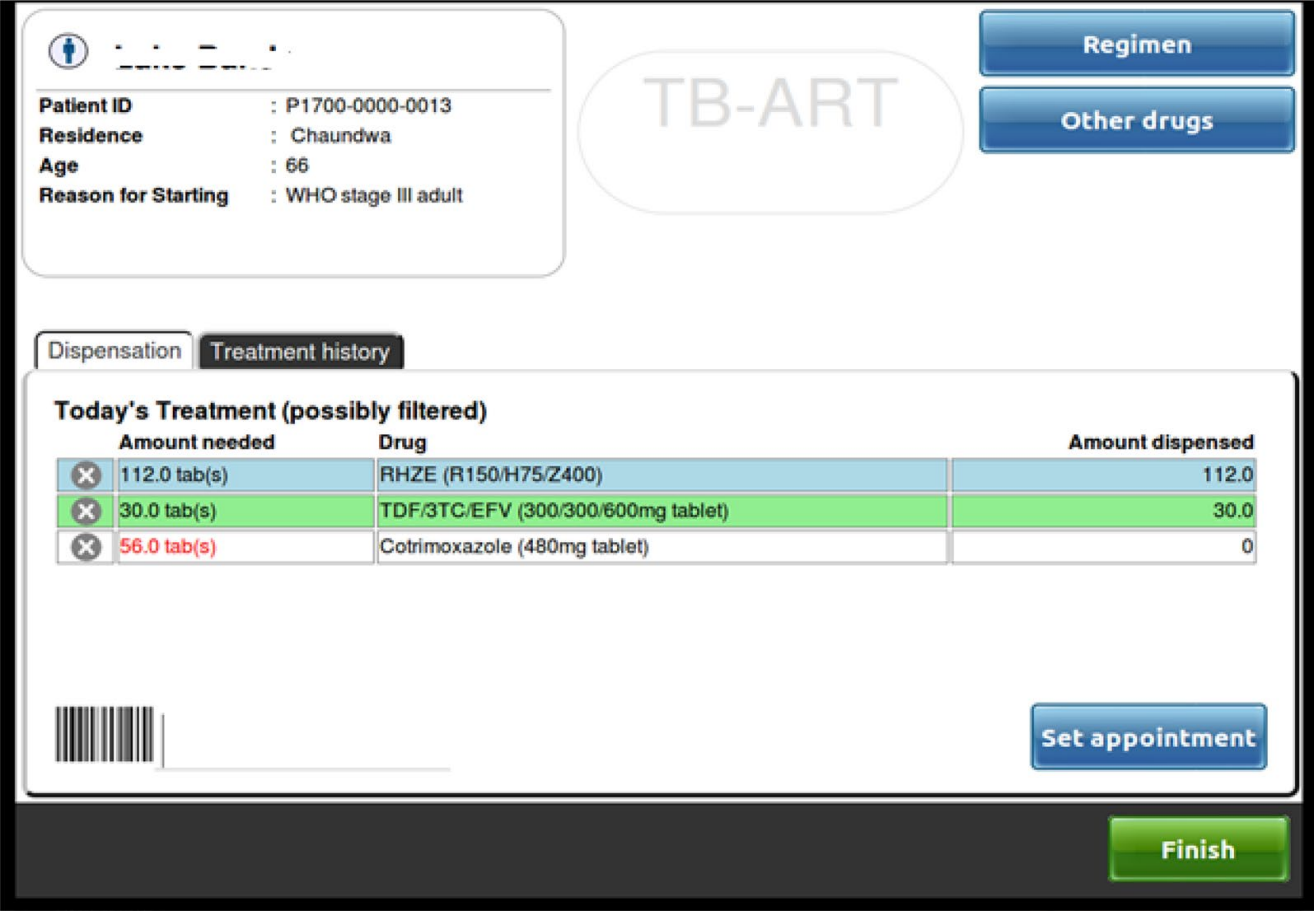
Drugs dispensing screen for the TB/ART Electronic Medical Record system at Martin Preuss centre in Lilongwe, Malawi

Drug dispensing with multiple regimens, including those for TB and HIV can become difficult and riddled with errors in stock management. In the TB/HIV EMR system, the drug dispensing process uses the same barcode scanning technology employed for patient IDs. Unique standard barcodes for different drug regimens and quantities are pre-printed on labels and placed in the pharmacy. For each patient receiving TB and/or ART drugs, pharmacy technicians scan the corresponding barcode for the prescribed drugs and quantities (Fig. 3). This technology may help reduce medication dispensing errors and improve drug accountability through this dual-check system of electronic scanning and pharmacist check.

#### Providing next appointment date and visit summaries

**Fig. 4:**
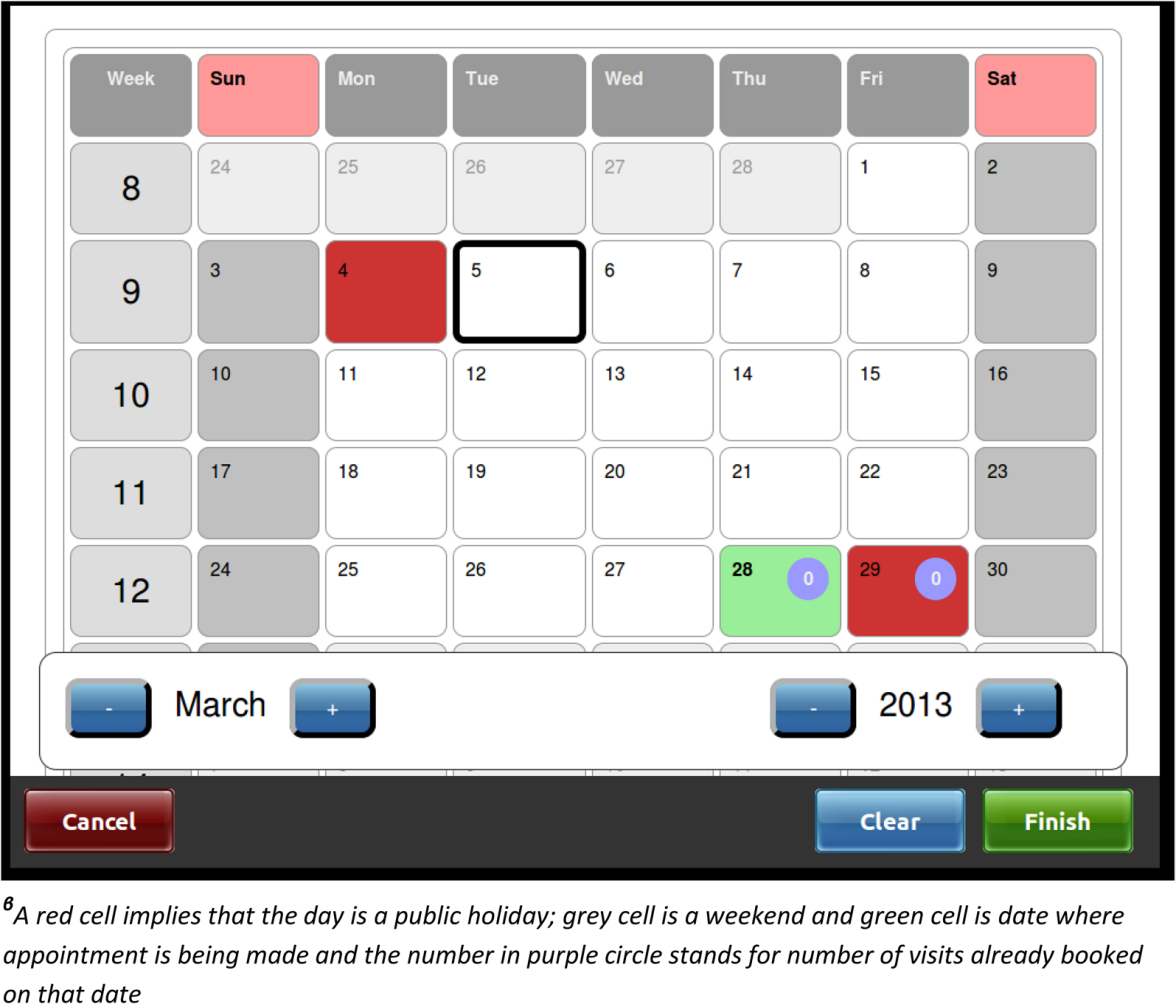
A screen for clinic appointment in a calender format for the TB/ART Electronic Medical Record system at Martin Preuss centre in Lilongwe, Malawi^β^

**Fig. 5:**
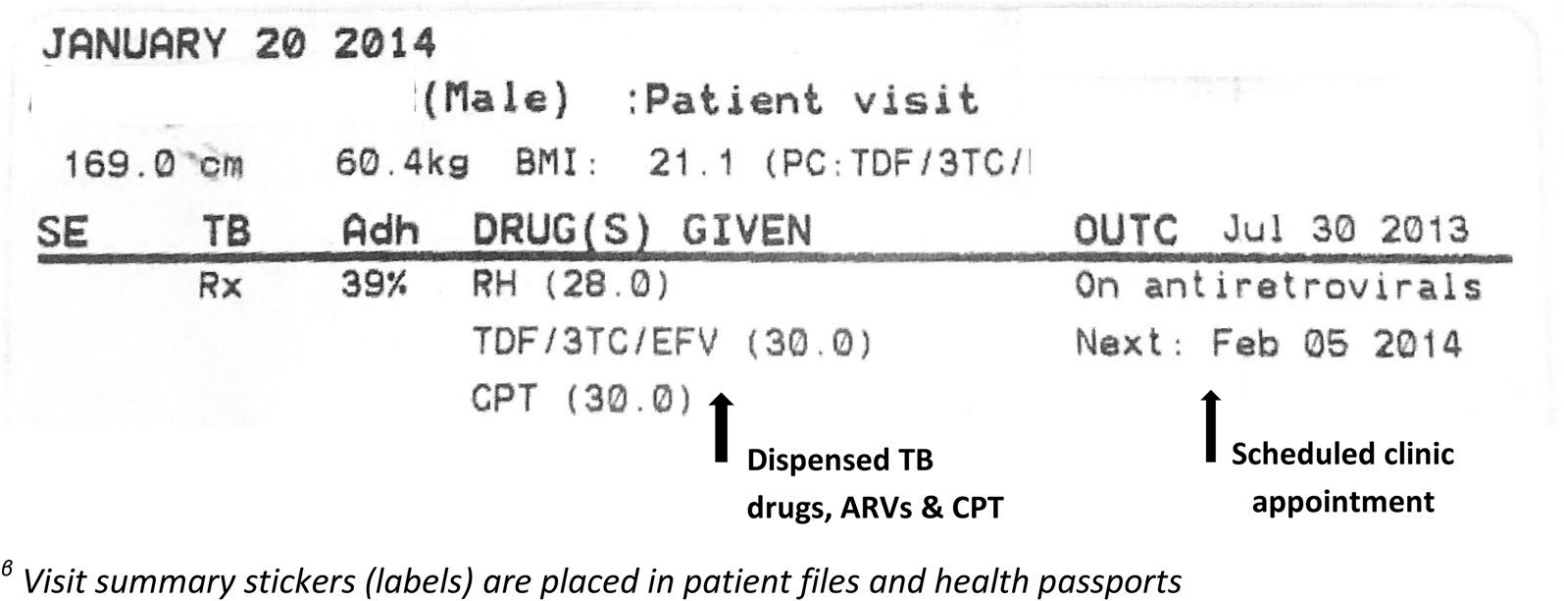
Duplicate visit summary stickers for the TB/ART Electronic Medical Record system at Martin Preuss centre in Lilongwe, Malawi^β^

Determination of next appointment date requires consideration of each patient’s drug requirements, adherence, and clinical review schedule—a process that is hard to do correctly by hand. In the TB/HIV EMR system, the system automatically calculates and presents the next appointment date in a calendar-format for provider confirmation based on the drug quantities dispensed and adherence profile, allowing the provider to change the date based on clinical or patient need (Fig. 4). At the end of each drug-dispensing visit, the system prints two summary labels placed in the patient health passport and paper file as a back-up. The summary includes ART status, current symptoms, drugs dispensed and next appointment date (Fig. 5).

#### Improving confidentiality through restricted access and user rights

The primary TB/HIV EMR system users are TB clinical officers, nurses, clinic clerks and health surveillance assistants. Access rights for specific tasks are assigned according to cadre and system management level. For instance, the clinic clerk cadre has access to registration and lab information modules only and cannot access tasks assigned to clinicians. A TB clinician trained in ART has access to all tasks through TB/HIV patient clinical review and prescription while a pharmacy technician is only allowed to dispense drugs. Super-users (expert system users at the clinic such as key monitoring and evaluation staff) and system developers have access to edit patient data including amending current and previous patients’ visit records. System usage logs record usernames of all users who enter or change patient data records for quality assurance and supervision purposes.

### Phase III: System testing and implementation

#### Pre-testing system components with key clinic users

As components of the system were being developed, a series of provider-focused pre-testing sessions were organized with mixed groups of up to 10, total, clinicians, clerks, and data staff to assess clarity and order of questions, and to verify compliance to national guidelines. We also tested various patient flow scenarios to ensure skip patterns were followed, e.g. family planning questions were not asked to young children or assessment of ART eligibility in confirmed HIV-negative patients. Also, pre-test sessions ensured that system alerts designed to remind clinicians to conduct HIV tests, order follow-up lab tests, consider drug contraindications, etc., worked as expected. Early pre-testing of TB/HIV EMR question flow and content helped system users understand the questions, identify missing or misplaced content, and avoid errors in misinterpretation.

#### On-site system training and preparation

After successfully pre-testing the system off-site, an intensive, two-day practical training was provided to approximately 20 healthcare providers and support staff at MPC—those with and without previous EMR experience. This training session was held over a weekend so that all staff could attend without affecting patients. Service providers first practiced specific tasks (e.g. registration, vital signs, TB results entry, ART initiation) according to their cadre (clerk, nurse, clinician, etc.) using touchscreen computers. Subsequent TB/HIV EMR practice activities mimicked diverse patient visit types requiring system users to “move” patients from registration through various clinic review services to drug dispensing. As system developers also attended the training, many issues in patient management and flow identified by users on Saturday could be immediately addressed by code fixes for further review and testing on Sunday. More complicated coding issues required additional days to correct and follow-up test with a smaller group of staff. Subsequently, through the initial on-site testing processes, system users identified various additional bottlenecks and system inconsistencies that required the developers to further refine system before a full clinic-based launch. After each system revision, additional, brief hands-on trainings were held on site with users to gain confidence and provide further feedback. When the system was completed, another weekend training was held with all clinic staff to demonstrate the final system and allow users to gain confidence in the system.

### System launch results

As an indication of the volume of data that can now be more easily accessed for monitoring and evaluation, in only our first 4 months of full system implementation, from April and July 2013, 787 individuals were registered in the TB/HIV EMR system: 369 (45 %) TB suspects and 418 (55 %) TB patients (Table 1). Among 369 TB suspects, 331 (90 %) had known HIV status and 177 (53 %) were positive. There were 149 (36 %) females among TB patients; the majority were adults aged 15 years and above. HIV ascertainment was 97 % among TB patients; 66 % were HIV positive. Of the TB patients with positive HIV test, 129 (48 %) were already on ART at TB registration, 106 (39 %) started ART while on TB treatment and 34 (13 %) had not started ART as end of July, 2013.

**Table 1:** Characteristics of TB Suspects and TB patients registered in the TB ART system between April and July 2013 at Martin Preuss Centre

Key programmatic indicators		N	%
TB suspects			
1.a	Total TB Suspects registered	369	
1.b	TB Suspects with known HIV Status	331	90
1.c	TB Suspects with known HIV positive	177	53
1.d	TB Suspects tested before registration	134	40
2.a	TB Suspects, sputum samples ordered	361	98
2.b	TB Suspects, sputum samples submitted	100	28
2.c	TB Suspects, sputum samples results	96	96
2.d	TB Suspects, sputum samples positive	18	19
2.e	TB Suspect, culture ordered	1	0
TB registration			
3.a	Total TB patients registered	418	
3.b	TB patients with known HIV status	406	97
3.c	TB patients with known HIV positive	269	66
Gender			
4.a	Female	149	36
4.b	Male	269	64
Age at registration			
5.a	TB patients 0–4 years at registration	4	1
5.b	TB patients 5–14 years at registration	17	4
5.c	TB patients 15 years or older at registration	397	95
TB category			
6.a	New Cases	381	91
6.b	Relapse	13	3
6.c	Retreatment After Default	1	0
6.d	Failure	1	0
6.e	Other	22	5
TB classification			
7.a	Pulmonary	320	77
7.b	Extra-pulmonary	98	23
DOT option			
8.a	Guardian	406	97
8.b	Hospital	9	2
8.c	HC	3	1
ARV status			
9.a	Before TB treatment	129	48
9.b	While on TB treatment	106	39
9.c	Not on ART during TB^a^	34	13

^a^ Some HIV/TB patients are still on TB treatment

### Lessons learned

#### Unexpected delays in software development and implementation

We experienced several unforeseeable implementation challenges on the software side. First, we underestimated the complexity of the software development process. Instead of the 1 year proposed from receipt of funding through implementation, we needed more than 2.5 years to complete the iterative cycle of pre-testing and revision process with all key stakeholders. In part, these delays were due to new national ART/PMTCT guidelines issued by the Malawi Ministry of Health launched in July 2011 [11] that necessitated significant revisions more than 6 months after project on-set. Further delays were caused by the inclusion of additional clinic services at a later stage, such as HIV testing and counseling services; these workflow changes took several months from OpenMRS coding through site-based testing and launch. Although inclusion of these additional patient services in the TB/HIV EMR led to delays, we chose this path because it would ultimately ease clinic flow and improve patient management. Second, we encountered some personnel issues that slowed implementation. Some healthcare workers did not attend the training while there was some turnover in TB clinic staffing, leading to bottlenecks in some clinic locations and the need for additional one-on-one training. Lastly, although the OpenMRS data model is flexible for software modification, advanced database management skills are required to access the data outside of routine reports, reducing the ability of MPC data staff to investigate additional patient care issues. Currently, Baobab Trust are working on extraction tools that can easily be used by data managers to generate customized reports and retrieve additional longitudinal data for monitoring and research.

#### On-site system deployment obstacles

Even with proper planning and successful pre-tests, disruptions in network connections, incorrect flow of questions, hardware issues, and challenges in the data transfer from paper to electronic still presented challenges during initial, on-site system deployment. To reduce the severity of these potential problems, we completed two key system checks before launch: (1) system intranet and terminals were tested and (2) a system dry-run from clinic registration to drug dispensing was conducted using diverse imaginary patients to identify and rectify additional issues before live deployment. Still, we encountered problems that required software developers to provide additional on-site assistance to avoid challenges in data quality while providers gained TB/HIV EMR skills and confidence.

#### Security concerns

EMR systems require strict data security protocols to ensure both patient confidentiality and data privacy. The TB/HIV EMR system has several built-in security features: (1) each user (service provider, system developer, IT support team etc.) has a unique username and password for logging in; (2) each user or cadre is restricted to their specific domain (e.g. service area); (3) only select “super users” may edit or revise patient entries; (4) patient information accessed by providers is restricted to the current patient (through entering patient’s unique ID or scanning its barcode); (5) data are stored in a password-protected, database and are accessible only by highly-trained internal staff; (6) the TB/HIV EMR system keeps an audit trail by tracking users, locations and timestamps for all data changes; and (7) data is encrypted during off-site data back up using secure encryption protocols.

#### Costs and sustainable funding

Securing start-up and sustainable funding are critical for system initiation and maintenance. Financial requirements were categorized into two groups; start-up and ongoing costs. Start-up (one-time) costs included purchasing hardware and developing software (Additional file 3: Table S1). The cost of a single work station for a healthcare provider was approximately US$1400; approximately 11 stations were required at MPC including two extras for backup. The personnel cost for developing the software was approximately US$39,000. With other server hardware, implementation and administrative costs, the total cost of the system was approximately US$103,000.00. Personnel costs of key staff and data personnel at Lighthouse Trust were provided as part of routine work salaries or from other funders, as acknowledged. Long-term sustainability of an EMR system requires hardware maintenance, software modifications, potential systems upgrades and paper patient labels on which clinic visit summaries are printed with an approximate quarterly cost of US$3,000. Funding for minor, yet ongoing, EMR system maintenance costs is provided by Centers for Disease Control and Prevention without which the sustainability of the systems and clinics would be challenging. It is hoped that the Ministry of Health will assume some of these costs in the future.

## Conclusions

Although implementation of large TB/HIV EMR system is complex, our three-phase approach to planning, design, and implementation allowed us to successfully launch the TB/HIV system in MPC clinic due to several key factors. First, an extensive, highly-participatory planning period with stakeholders ranging from service providers to MoH officials before and during the system development process, resulting in generating provider buy-in and ownership by the NTP who plan to adapt the system for national use. Second, alerts to order lab samples encourage high quality clinical practices while maintaining user-end simplicity, ensuing that providers were reminded to include all components of patient care even when faced with complicated patient management. Features such as prompts to order lab sample have the potential to decrease disparities in patient care because system-decision support is given to all providers. Third, working to ensure a consistent power-supply with a mix of electrical power and reliable backup system helped avoid unnecessary system failures and lost data. Fourth, a well-functioning paper-based TB registry greatly aided adoption of the TB/HIV EMR system because the clinic and all service providers already understood the importance of accurate and quality data in patient management. Lastly, adequate time should be allocated to mapping patient flows, assessing clarity and order of questions, development of software development and pre-test sessions to avoid unplanned delays and maintain user confidence.

Overall, the TB/HIV EMR system demonstrates the potential to enhance integration of TB/HIV services and facilitate improved decision-making among providers. We plan to further evaluate the data collected to inform service integration, strengthen monitoring and evaluation, and, ultimately, improve patient treatment outcomes. In the meantime, the system will be scaled up to other high-burden TB/HIV centers in Malawi for additional testing and evaluation at the national level. We believe the additional benefits of the TB/HIV EMR system towards improving patient care will become apparent as the system is expanded to other clinics and clinicians over time. Despite the personnel, financial, and technical investments required for success, other clinics, organizations, and public healthcare service providers should consider TB/HIV EMR systems as a beneficial investment in patient care.

## Additional files

10.1186/s13104-016-1943-4 Timeline for implementation of TB/HIV EMR.
10.1186/s13104-016-1943-4 Illustrative patient flows for common patient scenarios.
10.1186/s13104-016-1943-4 Approximate costs of TB/ART system development.
