# Supplementary material for: Developing a point-of-care electronic medical record system for TB/HIV co-infected patients: experiences from Lighthouse Trust, Lilongwe, Malawi
Source: BMC Res Notes. 2016 Mar 5;9:146. doi: 10.1186/s13104-016-1943-4 (PMC4779573; doi:10.1186/s13104-016-1943-4)
Supplement: Supplementary file 1 — 10.1186/s13104-016-1943-4 Timeline for implementation of TB/HIV EMR. [file 13104_2016_1943_MOESM1_ESM.docx]

**Figure 1: Timeline for implementation of TB/HIV EMR integrated electronic medical record system**

| Phase | Activity | Lead Org. | 2010 | | | | 2011 | | | | 2012 | | | | 2013 | | | |
| --- | --- | --- | --- | --- | --- | --- | --- | --- | --- | --- | --- | --- | --- | --- | --- | --- | --- | --- |
|  |  |  | Q1 | Q2 | Q3 | Q4 | Q1 | Q2 | Q3 | Q4 | Q1 | Q2 | Q3 | Q4 | Q1 | Q2 | Q3 | Q4 |
| One: Engaging multiple stakeholders | |  |  |  |  |  |  |  |  |  |  |  |  |  |  |  |  |  |
| 1 | Desk review: HIV and TB services at MPC and nationally | LH |  |  |  |  |  |  |  |  |  |  |  |  |  |  |  |  |
| 2 | Meetings: TB/HIV clinical staff and national TB/HIV programme | LH |  |  |  |  |  |  |  |  |  |  |  |  |  |  |  |  |
| 3 | Grant writing and awards | LH & BHT |  |  |  |  |  |  |  |  |  |  |  |  |  |  |  |  |
| 4 | Design general patient flows | BHT |  |  |  |  |  |  |  |  |  |  |  |  |  |  |  |  |
| 5 | Review patient flow with ART & TB clinic staff | BHT |  |  |  |  |  |  |  |  |  |  |  |  |  |  |  |  |
| 6 | Presentation of patient flow to LH and DHO for approval | BHT |  |  |  |  |  |  |  |  |  |  |  |  |  |  |  |  |
| 7 | Develop general (typical) clinical scenario flow diagrams | BHT |  |  |  |  |  |  |  |  |  |  |  |  |  |  |  |  |
| 8 | System requirements drafted & submission to MoH |  |  |  |  |  |  |  |  |  |  |  |  |  |  |  |  |  |
| Two: Developing TB/HIV EMR system components | |  |  |  |  |  |  |  |  |  |  |  |  |  |  |  |  |  |
| 8 | Finalize system requirements (software & hardware) | BHT |  |  |  |  |  |  |  |  |  |  |  |  |  |  |  |  |
| 9 | Finalize specific clinical scenarios (including rare cases) | BHT |  |  |  |  |  |  |  |  |  |  |  |  |  |  |  |  |
| 11 | Software specifications writing | BHT |  |  |  |  |  |  |  |  |  |  |  |  |  |  |  |  |
| Three: System testing and implementation | |  |  |  |  |  |  |  |  |  |  |  |  |  |  |  |  |  |
| 12 | System testing & refinement | BHT |  |  |  |  |  |  |  |  |  |  |  |  |  |  |  |  |
| 13 | Approvals for software specification | BHT |  |  |  |  |  |  |  |  |  |  |  |  |  |  |  |  |
| 14 | System deployment | BHT |  |  |  |  |  |  |  |  |  |  |  |  |  |  |  |  |
| 15 | System pilot & refinement | BHT |  |  |  |  |  |  |  |  |  |  |  |  |  |  |  |  |

LH: Lighthouse; BHT: Baobab Health Trust; DHO: District Health Officer; MoH: Ministry of Health
