# Supplementary material for: Developing a point-of-care electronic medical record system for TB/HIV co-infected patients: experiences from Lighthouse Trust, Lilongwe, Malawi
Source: BMC Res Notes. 2016 Mar 5;9:146. doi: 10.1186/s13104-016-1943-4 (PMC4779573; doi:10.1186/s13104-016-1943-4)
Supplement: Supplementary file 2 — 10.1186/s13104-016-1943-4 Illustrative patient flows for common patient scenarios. [file 13104_2016_1943_MOESM2_ESM.docx]

# Supplementary figure 2. Illustrative patient flows for common patient scenarios at Martin Preuss Centre in Lilongwe, Malawi

1. **TB suspects during sputum submission**

TB suspect arrives

TB suspect have HIV test results of within past 3 months?

TB officer captures TB suspect Demographics in chronic cough register

No

Yes

HTC carries out HIV test

TB suspect collects results

TB officer records HIV status in chronic cough register and give the TB suspect 3 sputum bottles

Next day TB suspect brings back the sputum bottles

2 or 3 days after sputum submission TB suspect collects results from the TB officer. TB officer updates chronic cough register

Lab carries out sputum test: 2-3 day response time for results

Sputum bottles taken to lab

Lab results to TB officer

Smear positive?

No

Yes

TB reception

TB suspect goes to for

chest X-ray examination

1. **TB suspect with X-ray results only**

TB Reception

HTC status

HIV test at HTC

TB +ve

TB Patient registers

at TB registration

TB patient collects TB drugs at TB Pharmacy

Home

The Clinician interprets the x-ray results and gives patient TB status

No

Yes

Not available/unknown

Available

1. **TB Patient with smear positive results**

TB reception

Patient sick

Yes

Clinician diagnoses patient

TB Patient registers at TB registration room

TB patient gets drugs at TB Pharmacy

Home

No

HTC status

Patient gets HIV tested at HTC

Available

Not available

1. **TB Patient during clinic follow-up visit**

Follow-up patient arrives

Need for Test at HTC

HTC counseling and HIV testing

Clinician diagnoses TB patient and changes drug prescription if there is need

Patient sick (side effects)

TB patient collects drugs at the TB Pharmacy

Home

No

Yes

No

Yes

TB officer updates TB patient treatment card and gives next appointment dates

1. **Transfer-in TB Patient during first clinic visit**

Transfer-in patient arrives

Need for Test at HTC

HTC counseling and HIV testing

Clinician diagnoses and prescribes different drug if patient has side effect

Patient sick (side effects)

TB patient gets TB drugs at TB Pharmacy

Home

No

Yes

No

Yes

TB officer updates TB patient treatment card and gives next appointment date

1. **ART initiation among TB patients**

New TB patient on ART arrives

TB officer opens ART file of patient at the TB reception

Staging, diagnosis and drug prescription by the clinician

Home

Patient attends ART session

Already went for ART session?

No

Patient gets drugs at ART Pharmacy

Yes

1. **Management of ART and TB services during follow-up visit**

TB patient on ART arrives

Patient gets ART file at the TB reception

Update patient ART visit, diagnose and drug prescription by the clinician

Patient gets drugs at ART Pharmacy

Home
