# Supplementary material for: Developing a point-of-care electronic medical record system for TB/HIV co-infected patients: experiences from Lighthouse Trust, Lilongwe, Malawi
Source: BMC Res Notes. 2016 Mar 5;9:146. doi: 10.1186/s13104-016-1943-4 (PMC4779573; doi:10.1186/s13104-016-1943-4)
Supplement: Supplementary file 3 — 10.1186/s13104-016-1943-4 Approximate costs of TB/ART system development. [file 13104_2016_1943_MOESM3_ESM.docx]

**Table S1. Approximate costs for the implementation of the TB/HIV electronic medical record system at Martin Preuss Centre in Lilongwe, Malawi**

|  | **Unit cost** | **Quantity** |  | **Amount (US$)** |
| --- | --- | --- | --- | --- |
| **Start-up** |  |  |  |  |
| **Hardware** |  |  |  |  |
| Workstation |  |  |  |  |
| Touchscreen | 710 | 11 |  | 7,800 |
| Label printer | 360 | 11 |  | 3,960 |
| Barcode scanner | 160 | 11 |  | 1,760 |
| Miscellaneous (e.g. cables, power sockets) | | |  | 170 |
| Server |  |  |  | 1,500 |
| Battery charge | 247 | 1 |  | 250 |
| Network switch | 110 | 1 |  | 100 |
| Battery (deep recycle) | 233 | 4 |  | 930 |
| Other network infrastructure |  |  |  | 24,530 |
| **System development** |  |  |  |  |
| Programmers |  | 5 |  | 39,000 |
| Project coordinator |  | 1 |  | 4,000 |
| Total |  |  |  | **43,000** |
| **Illustrative on-going implementation costs** | | |  |  |
| System orientation trainings |  | 2 |  | 7,000 |
| Labels for 6 months |  |  |  | 6,000 |
| IT support for six months |  |  |  | 6,000 |
| **Approximate total** |  |  |  | **103,000** |
